# HIRREM™: a noninvasive, allostatic methodology for relaxation and auto-calibration of neural oscillations

**DOI:** 10.1002/brb3.116

**Published:** 2013-01-14

**Authors:** Lee Gerdes, Peter Gerdes, Sung W Lee, Charles H Tegeler

**Affiliations:** 1 Brain State Technologies LLCScottsdale, Arizona, 85260; 2 Department of Neurology, Wake Forest School of MedicineWinston-Salem, North Carolina, 27157

**Keywords:** Allostasis, auto-calibration, biofeedback, electroencephalography, hemispheric asymmetry, HIRREM, insomnia, neural oscillation, relaxation, stochastic resonance

## Abstract

Disturbances of neural oscillation patterns have been reported with many disease states. We introduce methodology for HIRREM™ (high-resolution, relational, resonance-based electroencephalic mirroring), also known as Brainwave Optimization™, a noninvasive technology to facilitate relaxation and auto-calibration of neural oscillations. HIRREM is a precision-guided technology for allostatic therapeutics, intended to help the brain calibrate its own functional set points to optimize fitness. HIRREM technology collects electroencephalic data through two-channel recordings and delivers a series of audible musical tones in near real time. Choices of tone pitch and timing are made by mathematical algorithms, principally informed by the dominant frequency in successive instants of time, to permit resonance between neural oscillatory frequencies and the musical tones. Relaxation of neural oscillations through HIRREM appears to permit auto-calibration toward greater hemispheric symmetry and more optimized proportionation of regional spectral power. To illustrate an application of HIRREM, we present data from a randomized clinical trial of HIRREM as an intervention for insomnia (*n* = 19). On average, there was reduction of right-dominant temporal lobe high-frequency (23–36 Hz) EEG asymmetry over the course of eight successive HIRREM sessions. There was a trend for correlation between reduction of right temporal lobe dominance and magnitude of insomnia symptom reduction. Disturbances of neural oscillation have implications for both neuropsychiatric health and downstream peripheral (somatic) physiology. The possibility of noninvasive optimization for neural oscillatory set points through HIRREM suggests potentially multitudinous roles for this technology. Research is currently ongoing to further explore its potential applications and mechanisms of action.

## Introduction

This study introduces a novel, noninvasive electroencephalography-based interventional technology, called high-resolution, relational, resonance-based, electroencephalic mirroring (HIRREM™), or Brainwave Optimization™. The purpose of HIRREM is to facilitate relaxation and auto-calibration of neural oscillations through dynamic, auditory resonance with electroencephalic activity measured at high spectral resolutions. To contextualize HIRREM as an intervention with potentially multitudinous roles, in this section, we briefly review the array of diseases associated with neural oscillatory disturbance, share an overview of HIRREM and its development, and adduce the model of *allostasis* for explaining physiological regulation. Materials and Methods section describes procedures for provision of HIRREM. In Results section, data are presented from a clinical trial of HIRREM for individuals with insomnia, to illustrate a clinical application for HIRREM and associated changes in neural oscillatory symmetry.

### Disturbances of neural oscillation

Oscillation is a fundamental feature of physics and biology, and appreciation of the brain as a network of oscillators provides a highly integrative framework for understanding brain functionality (Buzsaki 2006). Neural oscillations can be impacted by stimuli which span a range of intensity from the subtle to the near lethal. Blast injuries sustained in warfare can produce disturbance of oscillatory synchrony (Sponheim et al. 2011). At the other extreme, weak signals can alter neural oscillations through the phenomenon of stochastic resonance (see HIRREM and EEG artifact or noise), whereby an increase in a neural system's noise level can, perhaps counterintuitively, enable the detection of an otherwise subthreshold periodic signal (Moss et al. 2004; McDonnell and Ward 2011).

Disturbances of synchronization of neural oscillation have been described in association with clinical disorders including epilepsy (Margineanu 2010), Parkinsonism (Gale et al. 2008), schizophrenia (Uhlhaas and Singer 2010), Alzheimer's disease (Dauwels et al. 2010), autism (Isler et al. 2010), and insomnia (Marzano et al. 2008). At the level of the cerebral hemispheres, oscillatory disturbances may manifest as imbalances of left–right EEG symmetry. Frontal EEG asymmetry has been described as a marker for affective style, with left and right frontal cortex associated with approach and withdrawal tendencies, respectively (Davidson et al. 1990). Other reports have associated hemispheric oscillatory asymmetry with posttraumatic stress disorder (Rabe et al. 2006; Engdahl et al. 2010), insomnia (St-Jean et al., 2012), attention-deficit disorder (Hale et al. 2010), autism (Stroganova et al. 2007; Lazarev et al. 2010), dyslexia (Spironelli et al. 2008), and schizophrenia (Swanson et al. 2010). Whether there could be a physiologic disturbance common to these asymmetries has not been much considered, but the hemispheric lateralization of management of the autonomic nervous system functioning (Yoon et al. 1997; Avnon et al. 2004; Craig 2005) – sympathetic and parasympathetic divisions by the right and left hemispheres, respectively – seems to raise the possibility that hemispheric oscillatory asymmetry may be an indicator of dysregulation of autonomic nervous system functioning.

Given that neuronal populations oscillate over a range of low to high frequencies, it is also possible to describe neural oscillatory disturbances as suboptimal proportionation of spectral EEG power across those frequency ranges, usually discerned through comparison of average amplitudes of broadband EEG ranges (i.e., delta, 0.5–4 Hz; theta, 4–8 Hz; alpha, 8–12 Hz; beta, 12–30 Hz; gamma, >30 Hz). Attention-deficit spectrum disorders (Barry et al. 2003), mild cognitive impairment (Babiloni et al., 2010), dementia (Dauwels et al. 2010), and traumatic brain injury (Moeller et al. 2011) have been associated with relative excess power in low frequencies (i.e., delta and/or theta) in comparison with high frequencies. Other forms of suboptimal proportionation of spectral EEG power have been reported with insomnia (Perlis et al. 2001; Wolynczyk-Gmaj and Szelenberger 2011), alcoholism (Campanella et al. 2009), and chronic fatigue syndrome (Decker et al. 2009).

The existence of an array of conditions which share thematic forms of neural oscillatory disturbance – asymmetry and suboptimal proportionation of spectral power – suggests that a positive role may exist for technologies that may constructively impact neural oscillations in the direction of greater symmetry and optimized proportionation.

### Definition and development of HIRREM

Through serendipity, one of the authors of this article (L. Gerdes) found that near real time reflection of neural oscillatory activity back to the brain through the medium of audible sound appeared to facilitate a state of relaxation wherein the brain, itself, would tend to change its own activity patterns toward greater hemispheric EEG symmetry and more optimized proportionation of regional spectral EEG power. We thus described the process facilitated as one of relaxation and auto-calibration for neural oscillations. The methodology has been continuously refined since its development in 2000–2002, and since 2010, it has been described technically as high-resolution, relational, resonance-based electroencephalic mirroring or HIRREM. The technology is based on provision of auditory musical tones corresponding to dominant frequencies detectable in individual spectral EEGs, to permit resonance between neural oscillatory frequencies and auditory tones. It requires no direct energetic input to the brain, no cognitive guidance or education from a clinician, nor any referencing against population norms for the EEG.

### Allostatic regulation of neural oscillations through HIRREM

Because of the variety of conditions, including “somatic,” that have been reported to benefit from HIRREM on an anecdotal basis (see Overview section), we infer that HIRREM facilitates self-guided and healthful reorganization of neural oscillations at some level(s) of primary neural process, with consequences for both neuropsychiatric health and downstream peripheral physiology. To model the larger theoretical role of HIRREM, we adduce the concept of *allostasis* as defined by Sterling (2004, 2012). Allostasis refers to stability (stasis) through change (allo). Allostasis highlights the centrality of the brain as the master control center for human physiology, whose primary function is to serve as an instrument for optimal predictive regulation. The concept of allostasis may be clarified through comparison with the more commonly used biomedical concept of homeostasis.

#### Homeostasis as a model of physiological regulation through maintenance of predetermined and normative set points

Homeostasis refers to stability (stasis) through constancy (homeo) and is a model of physiological regulation in which various systems are described in terms of their requirement to maintain various set points at constant values. These values are deemed normative, and systematic deviations are generally considered disease states. The objective of biomedicine is to identify the mechanisms underlying regulation of set points. The guiding assumption is that *mechanisms are dysfunctional* in states of disease and therefore are the cause of deviated set points. Therapeutics thus consists of intervention to correct dysfunctionality of local mechanisms. The system set points being “defended” in homeostasis are typically defined based on prespecified level of demand, calculated on norms derived from historical or other controlled influences. Homeostasis thus focuses on functionality (or dysfunctionality) of local mechanisms without a nuanced appreciation for how complex environmental contexts drive system needs or set points in the first place. The seeds for the concept of homeostasis were developed before the dissemination of evolutionary theory (Sterling 2012), and thus, the homeostasis model reflects an understanding of life itself as being fundamentally unchanging.

#### Allostasis as a model of brain-guided predictive regulation through dynamic optimization of system set points

Allostasis conceives the brain as the master regulator which, when well-functioning, anticipates changing needs in a constantly changing environment and recalibrates system set points in accordance with present or anticipated demands. The brain dynamically allocates and re-allocates the body's energetic resources in order to *optimize fitness*. In the Sterling model of allostasis, activities of the present should meet the needs of the present; they should not be organized to meet the demands of the past or other nonsalient norms; and they must also include anticipation and preparation for the needs of the future. In the allostasis model, deviations of system set points may be indicative of disease states, but local mechanisms are not viewed as being intrinsically dysfunctional – rather they are simply responding to a different level of demand.

**Figure 1:**
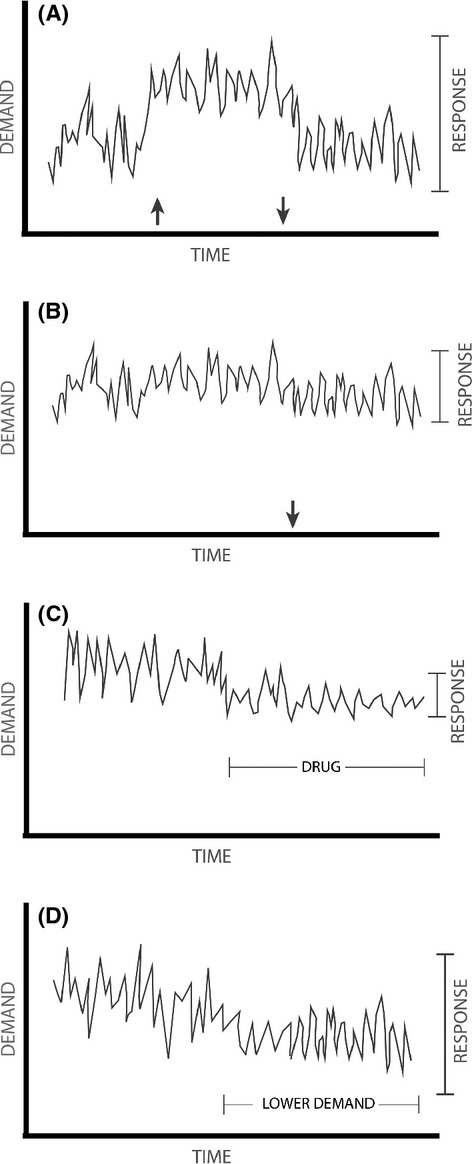
Rhythmic output of a model system under conditions of health and fluctuating demand (A); “stuckness” due to prolonged or possibly acutely potent demand (B); pharmacotherapy (C); and an idealized health intervention, associated with gradual reduction of demand (D). Black vertical arrows denote changes in demand on the system. Adapted from Sterling (2004).

Figure 1 (adapted from Sterling 2004) illustrates a simplified general model for how a healthy system will adjust its output set points to respond dynamically for the changing levels of ambient demand (Fig. 1A). The system set point can become stuck (Fig. 1B), for example, because of an acutely potent demand or elevated demand over time (e.g., a trauma or chronic stress), to produce outputs which are calibrated for the historical level of demand, despite the emergence of a new and lower level of demand. Pharmacological therapy (Fig. 1C) can alter and clamp the system set point at an output level which appears more congruent with the present demand, but at the expense of depriving the system of its dynamic range of action. An ideal intervention (Fig. 1D) would encourage a diseased system to relax, become “unstuck,” and recalibrate output for the true and present (not historical) level of demand.

Allostasis would appear to be consistent with the view of physiology from nonlinear dynamical theory, which considers system complexity to be a hallmark of health (Goldberger et al. 2002). It may be that it is the loss of complexity, rather than the loss of regularity, which is associated with disease states. Decreased neural functional complexity has been described in Alzheimer's disease (Jeong 2004), mild cognitive impairment (Cantero et al. 2009), posttraumatic stress disorder (Chae et al. 2004), and autism (Bosl et al. 2011; Catarino et al. 2011). Decreased EEG complexity can be observed in epileptic seizure (Babloyantz and Destexhe 1986), and increased variability of synchrony has been shown to be associated with recovery from pediatric traumatic brain injury (Nenadovic et al. 2008). Increased complexity appears to be a normal and perhaps healthy feature of the EEG over the course of human development from infancy to older age (Meyer-Lindenberg 1996; Anokhin et al. 2000; McIntosh et al. 2008; Muller and Lindenberger 2012).

#### Allostasis and disease

**Figure 2:**
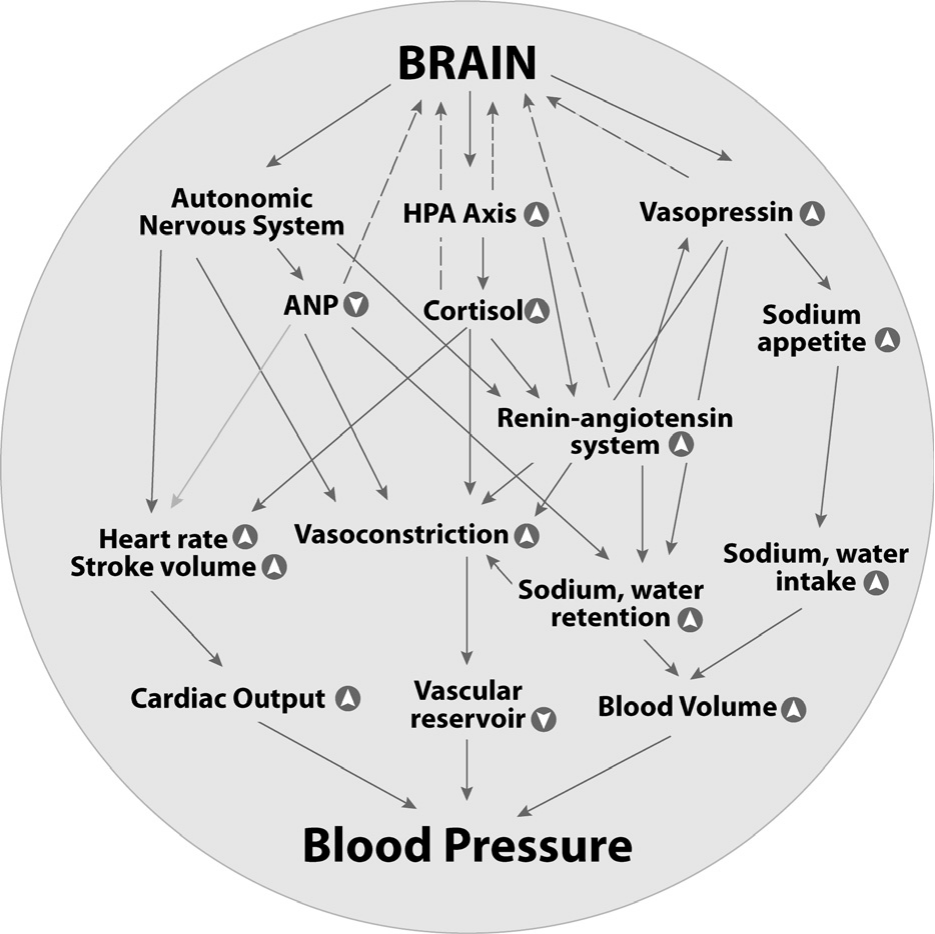
Allostatic model of blood pressure regulation (adapted from Sterling 2004).

The difference between the homeostasis and allostasis models of physiological regulation can be illustrated through the ways they explain blood pressure management (Sterling 2004). Homeostasis portrays blood pressure as a set point managed by blood volume, vascular resistance and cardiac output, and medical interventions aim to impact mechanisms related to the management of those variables. Allostasis portrays blood pressure as a set point influenced proximally by vascular resistance, volume, and cardiac output among other factors, but ultimately managed by the brain (Fig. 2). Under the allostasis model, the ultimate way for blood pressure to change is for the brain itself to adopt a different set point. Adoption of new (and changing) blood pressure set points that are more optimally calibrated for complex (and changing) environmental demands likely necessitates high-level integration of information at the level of the cortex.

The concept of allostasis has been especially developed to explain the deleterious effects of chronic stress on health (McEwen 1998, 2007). *Allostatic load* may manifest when otherwise helpful and adaptive neural response mechanisms, especially the response of the hypothalamus–pituitary–adrenal (HPA) axis to an environmental challenge, have been highly activated over time. For example, circulation of effectors related to the HPA axis including cortisol, epinephrine, and norepinephrine may be helpful in the setting of an acute stressor, but their extended presence (weeks, months, or years) may cause damage to the tissues they would otherwise protect. Allostatic load may explain the relationship between low socioeconomic status and poor health outcomes (Seeman et al. 2010).

Various other health and disease phenomena have also been re-contextualized with the model of allostasis and allostatic load, including migraine (Borsook et al. 2012), sleep deprivation (McEwen 2006), glucose regulation (Stumvoll et al. 2003), fibromyalgia (Martinez-Lavin and Vargas 2009), perinatal health outcomes (Shannon et al. 2007), aging (Karlamangla et al. 2006), asthma (Bahreinian et al. 2012), nonalcoholic fatty liver disease (Baffy 2012), substance abuse (Koob and Le Moal 2001; George et al. 2012), and bipolar disorder (Kapczinski et al. 2008).

#### HIRREM as a precision-guided technology for allostatic therapeutics

Allostatic therapeutics is a field yet to be systematized. Nonetheless, it stands to reason that allostatic therapeutics will invoke, for example, the need for multicomponent and behavioral interventions (e.g., Ornish et al. 1998; Loizzo et al. 2009; Streeter et al. 2012) which are intended to change demand levels, so that neural functioning can recalibrate toward more healthful set points in subject-specific increments. Yet, considering the inertia often associated with these domains, the objectives of allostatic therapeutics may be more effectively realized if the brain itself is facilitated to calibrate its oscillations to desirable set points. Thus, HIRREM technology may be well suited to serve as a catalyst for neural changes underpinning healthful behavior change.

## Materials and Methods

### Overview of HIRREM requirements and application

The physical requirements for provision of HIRREM include a standard PC-based desktop or laptop computer, a specialized EEG amplifier and preamplifier, EEG sensors, standard earbud headphones, a specialized software program, and a reclining chair. EEG sensors connected to the preamplifier (powered by a 9 V, 400 mAh rechargeable lithium ion battery) filter 50 and 60 Hz activity so as to reduce the contribution of environmental electromagnetic noise. Sampling rate is 256 Hz. The amplifier is powered by a standard Windows-based laptop computer and uses a 16-bit A/D converter with a notch filter for rejection of signal >50 dB at 50 or 60 Hz. Signal processing is done in a 64-bit computer processor. Technologists are trained to identify EEG evidence of grossly recurring artifact (e.g., eyeblinking) or sensor displacement from the scalp, but the software does not attempt to identify artifact or other forms of noise (see HIRREM and EEG artifact or noise).

Provision of HIRREM for an individual consists of EEG and questionnaire-based assessment, active HIRREM sessions (generally 60–90 min each, 3–10 sessions or more), and software-supported data analysis by a technologist. Questionnaires capture data related to symptoms, medical history, and objectives for undergoing the HIRREM procedure. Data are collected in a master database (see below), which is used to help guide ongoing innovations of HIRREM technology. Based on clinical experience suggesting a deleterious effect on outcomes, subjects are strongly advised to abstain from alcohol and recreational drugs for the period of their HIRREM sessions and for at least 3 weeks thereafter.

### Procedure for HIRREM assessment

Assessment consists of serial measurements of two-channel EEG using active sensors, with scalp locations identified based on the International 10–20 EEG system. Two recording sensors, two reference sensors, and one ground sensor are used. Measurements are taken at homologous regions of the hemispheres (F3/F4, C3/C4, T3/T4, P3/P4, O1/O2) for eyes closed (1 min), partially closed (1 min), and eyes open (1 min), with subject in an upright, seated position. For eyes closed, subjects are asked to rest and relax quietly. For eyes open, subjects are given standardized tasks involving numerical digit-recall (F3/F4), reading silently (C3/C4), calculations (P3/P4), listening comprehension (P3/P4), and visual observation (O1/O2). A sixth measurement is taken along the midline of the scalp at FZ/OZ. The reference sensors are connected at A1/A2 and linked. The EEG portion of the assessment takes approximately 45–60 min to complete.

### Procedure for HIRREM exercises

With the subject comfortably at rest, sitting or reclining in a zero-gravity chair, sensors are placed over specific target areas on the scalp. As with the assessment, up to two recording sensors, two reference sensors, and one ground sensor are used. Most HIRREM protocols (defined as a combination of sensor montage and the specific software design) capture two channels of electroencephalic data between homologous regions of the hemispheres. Two-channel single-sided protocols may be used to focus attention on apparently recalcitrant oscillatory activity localizing in a particular region. One-channel protocols may also be used to focus attention, especially in “alpha” and “beta” frequency bands, on single regions without a particular interest in symmetry with the homologous region of the contralateral lobe.

Initial placements for the sensors are recommended by the HIRREM software based on cortical regions and spectral frequency ranges exhibiting the greatest asymmetries and/or suboptimal proportionations of spectral power, based on data collected during the assessment. Single HIRREM sessions generally consist of 5–8 protocols, each lasting 5–15 min. In general, sessions are provided on a relatively compressed schedule, that is, as intensively as two per day, or generally no more slowly than three per week, with 10 sessions typically being completed within 3 weeks. A typical HIRREM session lasts 60–90 min.

During all HIRREM protocols, subjects wear standard earbud headphones, through which they listen to the musical tones generated by the HIRREM software algorithms. Subjects are encouraged to relax in the zero-gravity chair at a near-prone angle so as to maximize cerebral blood flow, and they may be encouraged to visualize themselves in a peaceful setting in nature or simply to pay attention to their breathing. The majority of exercises take place with eyes closed. For exercises with eyes open, subjects may read a book or relax while watching changing graphics on a computer monitor.

### High-resolution spectral analysis of electroencephalic data and dynamic, iterative engagement of dominant frequencies

The HIRREM system includes proprietary preamplifiers and filters which allow collection of electroencephalic data to the nearest 0.01 Hz, at the level of the sensor attached to the scalp. HIRREM software analytics then identify dominant frequencies in specific spectral brackets, in up to 48,000 bins of spectral data for any given bracket. Brackets are assigned by the software based on a proprietary algorithm. The software compares the two channels of data to ascertain the symmetry between channels of EEG information and proportionation of spectral power within the channels. From the bracket of frequencies assigned for the subject's exercise, the HIRREM software translates the dominant EEG frequency in a given instant of time to an audible musical tone, which is received by the subject through earphones. Depending on algorithm calculations, the delay between measurement and analysis of neural oscillatory activity and consequent presentation of corresponding musical tones can be as narrow as an estimated 12 msec. The process then iterates.

The HIRREM mathematical algorithms to define specifically how and when the dominant EEG frequencies are selected for resonance are informed by relationships among the parameters of the individual's own unique spectral EEG. The specific tone is produced from a proprietary mathematical algorithm principally informed by the dominant frequency within the observed spectral bracket. A sample sequence of tones produced during 1 min of a HIRREM exercise and the corresponding notes on the pentatonic scale are available as Internet resources, in the form of audio, and pdf files.

Application of HIRREM exercises to the bilateral temporal lobes is emphasized, as we theorize that comparison of spectral EEG amplitudes in simultaneous recordings at the bilateral temporal lobes (T3 and T4 in the 10–20 International EEG system) may provide an opportunity to engage the degree of balance between the sympathetic and parasympathetic divisions of the autonomic nervous system. As noted in Introduction, numerous studies have found that management of the autonomic nervous system is lateralized in the cerebral hemispheres. Specifically, right insular cortex appears to drive sympathetic functioning, whereas left insular cortex drives parasympathetic functioning (Craig 2005). T3 and T4 are located over Brodmann areas 21 and 22, respectively, at the middle and superior gyri of the temporal lobes (Homan et al. 1987) and are therefore in the proximity of insular cortex. Apart from a focus on the temporal lobes, HIRREM exercises take place for major regions of the cortex including frontal, parietal, occipital lobes, central strip, and the midline, and across the EEG frequency spectrum in each of those locations.

**Figure 3:**
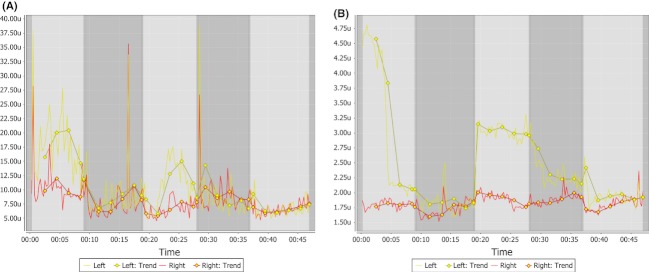
Changing asymmetry at the bilateral temporal lobes over the course of five successive HIRREM exercises, in the 0–1 Hz (A) and 36–48 Hz (B) frequency bands. Yellow line represents amplitudes at T3, and red line represents amplitudes at T4. Each HIRREM exercise is represented by a background with alternating shades of gray.

At the conclusion of a single HIRREM session, the provider runs an analysis program which shows summary data for the session. Comparative amplitudes and coherence of the two channels of data collected during various protocols can be evaluated in the following 10 broadband frequency ranges: 0–1, 1–3, 3–5.5, 5.5–7.5, 7.5–10, 10–12, 12–15, 15–23, 23–36, and 36–48 Hz. Cortical regions and spectral frequency ranges of interest can be chosen for subsequent HIRREM sessions. Examples of the output from this analysis program are shown in Figure 3, which depicts changing amplitudes in the 0–1 and 36–48 Hz frequency bands, over five successive HIRREM exercises at the temporal lobes. As of the time of this writing, new analytic software is being developed to enable computer-guided recommendations for protocols to implement in successive HIRREM sessions. The new session-to-session analytic tool performs primarily time-domain analysis of amplitudes in the 10 aforementioned ranges, aggregated over 15-sec intervals after removing the first and last 30 sec to eliminate artifacts related to the start or end of the exercise. Data are fitted using regression analysis to determine trends of symmetry and proportionation of spectral power. Based on the identified trends, HIRREM protocol suggestions are made for the next session.

### Use of HIRREM master database to guide iterative innovations in hardware and software

All data associated with the HIRREM procedure including responses to the questionnaires, the assessments, and all HIRREM exercises are stored locally on computers at various locations throughout the world where HIRREM is provided. These locations are linked by Internet to the corporate headquarters of the developers of HIRREM technology (Brain State Technologies, Scottsdale, AZ). On a nightly basis, these data are uploaded without personal identifiers into a master database located at the corporate headquarters.

The information in this database allows for exploratory hypothesis testing to identify possible correlations between symptom clusters and EEG patterns, thereby facilitating the refinement of HIRREM software designs and protocol options. Thus, HIRREM technology is continuously adjusted and refined to selectively provide resonance for cortical regions and EEG spectral ranges which may better assist the subject's own unique self-regulatory process. Notably, the master database is not used to generate normative values for EEG parameters, against which subjects would be compared and which would be held as a basis for therapeutic goals.

## Results

### Overview

As of September 2012, HIRREM technology is being used by over 200 providers in North America, Europe, South Africa, Asia, and Australia. Over 50,000 subjects have undergone HIRREM worldwide and are contained in the database. Case series of outcomes have been reported for individuals with neurodegenerative disease (Singh and Gerdes 2009a) and depression (Singh and Gerdes 2009b). Anecdotally, a range of benefits are reported including relief from sleep disorders, depressive symptoms and anxiety, reduced symptomatology related to trauma, improved cognitive functioning, relief from addictive urges, improvement in cardiovascular and gastrointestinal conditions, and others. A randomized, wait-list controlled pilot trial has shown efficacy of HIRREM for relieving symptoms of insomnia (Tegeler et al. 2012), and a placebo-controlled trial testing efficacy for migraine has been completed.

### Changes in temporal lobe EEG asymmetry associated with use of HIRREM as an intervention for insomnia

We present changes in temporal lobe asymmetry for 19 subjects enrolled in a randomized, wait-list, controlled pilot trial of HIRREM as an intervention for insomnia. Methods and main clinical outcomes for this study have been reported elsewhere (Tegeler et al. 2012). Mean age of subjects was 45 (70% women), and at baseline, mean score on the Insomnia Severity Index (Bastien et al. 2001) was 18.8, indicating, on average, clinical insomnia of moderate severity. Subjects also reported substantial depressive symptomatology (average CES-D score 14.9). All subjects underwent an average of nine (range 8–13) HIRREM sessions, beginning either immediately after enrollment into the study or after a waiting period (usual care) of 6 weeks. At the primary endpoint, subjects undergoing HIRREM reported a reduction of 10.3 points in the ISI, while those undergoing usual care reported no change.

Though HIRREM exercises were conducted at the temporal, occipital, parietal, central, and frontal lobes, and anterior and posterior midline, temporal lobes were chosen for the present analysis on an a priori basis, because of the proximity of the insula and limbic structures related to autonomic functioning (see High-resolution spectral analysis of electroencephalic data and dynamic, iterative engagement of dominant frequencies). Data for calculation of asymmetry scores were derived from the HIRREM exercise conducted at the bilateral temporal lobes, for each subject and for each session. For those sessions in which two exercises were conducted at the temporal lobes, the first exercise was used for calculation of the asymmetry score. Asymmetry scores were calculated based on the log of the average spectral power (23–36 Hz) at T4 over the course of the 8-min HIRREM exercise, minus log of the average spectral power (23–36 Hz) at T3. The high frequency (23-36 Hz) range of the EEG was chosen for the present analysis because of evidence of high-frequency arousal as being contributory to insomnia (Perlis et al. 2001; Wolynczyk-Gmaj and Szelenberger 2011).

**Figure 4:**
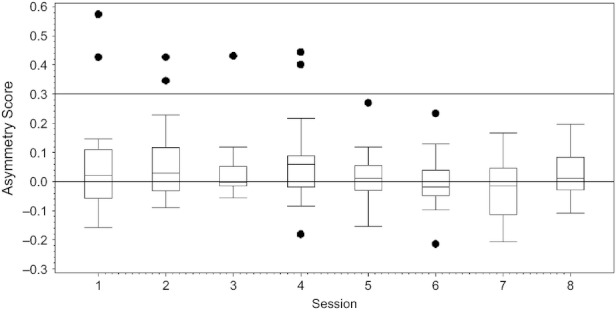
Tukey plot of average asymmetry scores between T3 and T4 in the 23–36 Hz range, over the course of eight successive HIRREM exercises for 19 subjects enrolled in a clinical trial evaluating efficacy of HIRREM as an intervention for insomnia. Whiskers extend to last observation not more than 1.5 times the interquartile range. Dots represent observations more than 1.5 times the interquartile range.

**Figure 5:**
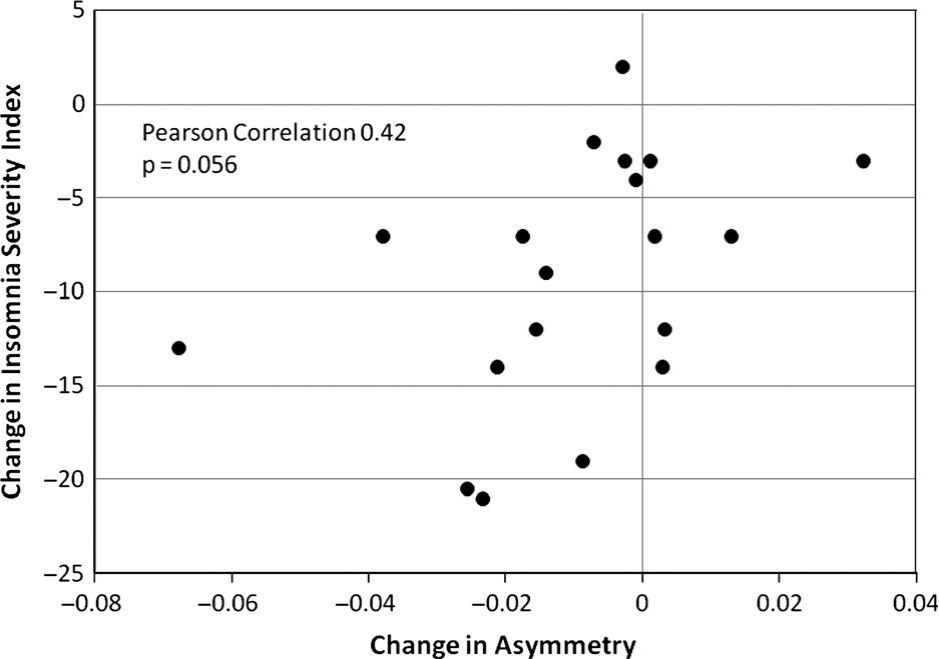
Correlation between individual change in asymmetry between T3 and T4 in 23–36 Hz range over eight serial HIRREM exercises, and change in insomnia symptoms as measured by change in ISI score. More negative change in asymmetry scores indicates lesser dominance of amplitudes at T4 over T3. More negative ISI change scores indicate greater reduction in insomnia symptoms. Change in asymmetry is calculated as the slope of a linear equation fitted to the series of asymmetry scores over eight serial HIRREM exercises, for each individual.

Figure 4 shows the average asymmetry score for T3 in comparison with T4, for all 19 subjects over the course of their HIRREM sessions. Rightward asymmetry (T4 > T3) diminished over the course of six HIRREM sessions, followed by a shift to average leftward asymmetry (T3 > T4) for session 7, and a return to rightward asymmetry for session 8. To test whether change in asymmetry was correlated with improvement in insomnia symptoms, an individual asymmetry change value was computed for each individual, by fitting a simple linear equation to the serial asymmetry scores of each subject. A positive slope for this equation indicated a trend of higher asymmetry scores over the course the sessions, or greater dominance of T4 over T3, whereas a negative slope indicated a trend of lower asymmetry scores, or diminishing dominance of T4 over T3. A plot of individual change of asymmetry scores against individual change in insomnia as measured by the Insomnia Severity Index (Bastien et al. 2001) is shown in Figure 5. There was a trend for reduction of temporal lobe high-frequency EEG asymmetry in the direction of less dominance of T4 over T3 to correlate with greater reduction of insomnia symptoms.

### Safety and side effects

HIRREM has been found to be a safe procedure. Based on experience with provision of case management support (by Brain State Technologies), feedback from clients and the HIRREM provider community, and three IRB-approved studies based at a tertiary medical center, the developers and researchers are not aware of any serious adverse events resulting from undergoing HIRREM.

On an anecdotal basis, individuals undergoing HIRREM may report an apparent “release of emotions” or paradoxical effects especially initially, which can manifest as brief periods of increased awareness of emotional states, both positive and negative. These experiences are typically transient, that is, lasting intermittently over the course of one to several days. In the course of provision of HIRREM to 118 subjects participating in three university-based IRB-approved studies, subthreshold changes in emotional symptomatology (not requiring additional clinical intervention or necessitating discontinuation of sessions) were estimated by the principal investigator to occur in approximately 5–10% of subjects. All HIRREM sessions are administered by technologists who have been certified in the procedure, including guidelines for addressing emotional releases that may occur. If emotional releases are prolonged or intense, individuals are advised to see a mental health professional for additional evaluation or treatment.

## Discussion

### Comparison with other interventions to remediate disturbances of neural oscillations

There is no absolute schema for the classification of interventions that can impact neural oscillations, nor is there a logical consensus for terminology. For example, while the term “neuromodulation” is used by some providers to refer specifically to surgically implanted devices for direct stimulation of neural tissue, there is no doubt that myriad other interventions can act as modulators of neural function either through stimulation of the cortex through the skull, pharmacological action (e.g., influencing neuronal membrane potentials or ion channel function), or sensory stimulation of the peripheral nervous system from consumer-oriented or computer-based technologies.

A variety of device-based interventions exist which can impact neural oscillations. Electroconvulsive therapy, transcranial magnetic stimulation, and transcranial direct current stimulation can impact neural oscillations through delivery of electromagnetic energy from a device to the cortex through the skull. Deep brain stimulation, transcutaneous electrical nerve stimulation, vagus nerve stimulation, and others impact neural oscillations by delivery of electrical impulses through implanted devices that are in direct contact with neural tissue. Stimulation devices available in the consumer marketplace can entrain neural oscillations toward specific and predetermined frequencies through delivery of rhythmic light and/or sound.

EEG operant conditioning (also known as EEG biofeedback or neurofeedback) is a collection of techniques for measuring neural oscillations in broadband EEG ranges and teaching individuals how to consciously increase or decrease the amplitudes in those ranges. Individuals are presented with visual and/or auditory stimuli as feedback when average amplitudes of the selected ranges cross a predetermined threshold. These stimuli are thus presented as either “rewards” for movement of energy toward the specified parameter or “inhibits” if the energy moves in the nonnormative direction. To set parameters of training, providers may access databases of EEG assessments to formulate a normative basis for evaluation and treatment. Clinical studies of EEG operant conditioning have been reported for a number of disorders including epilepsy (Sterman 2010) and attention-deficit disorder (Arns et al. 2009). The technology is noninvasive and generally considered a low-risk procedure with minimal side effects.

A limitation of EEG operant conditioning (conscious associative learning) to change average amplitudes of broadband spectral EEG ranges (i.e., delta, theta, alpha, beta, gamma) is that it is likely associated with collateral, nonuseful learning. That is to say, learning to change amplitudes across these broad ranges will entail learning to change amplitudes for some segments of those frequency ranges that are likely nonproblematic for that individual (Salansky et al. 1998). Relatedly, use of broadband EEG ranges entails a relative blurring of large quantities of EEG information. Szava et al. (1994) found that broadband analysis could obscure peaks of energy detectable in narrower frequency ranges that appeared to be associated with pathology. Also, the precise spectral location of the peak frequency for the alpha (8–12 Hz) range is variable across individuals, and the location of this peak is a meaningful parameter that has been correlated with development (Cragg et al. 2011) and cognitive performance (Angelakis et al. 2004). Engagement with an individual's unique spectral EEG fingerprint is not possible with technologies that rely on standard broadband EEG frequency ranges.

### HIRREM and EEG artifact or noise

Artifact identification and rejection are thematic to the field of EEG. EEG artifacts may include a variety of discrete phenomena including abnormalities of the EEG tracing which are due not to neural oscillation but rather to scalp muscular contraction, eyeblinking, or head or sensor movement.

For the practice of EEG operant conditioning, the identification of EEG artifact is mission-critical, because the presentation of reward or inhibit signals in response to peripheral muscular contractions (for example), rather than neuronal oscillations, is subversive to the purpose and basis of the enterprise. (Likewise, artifact identification is critical for medical EEG especially insofar as definitive diagnosis depends on accurate characterization of EEG waveforms which are abnormal but may manifest inconsistently.)

Because HIRREM technology does not aim to consciously teach the individual through signals of reward or inhibition, we postulate that there is little if any jeopardy associated with providing auditory signals which are informed by nonneural sources and are therefore “meaningless.” (Nor does HIRREM aim to diagnose disease.) Rather we infer that the brain responds to epochs of HIRREM sounds generated from grossly noisy EEG artifact in the way that it would respond to grossly noisy sounds. Furthermore, artifact-associated data will tend to be distributed symmetrically, and because HIRREM algorithms are based on the relationship of activity between homologous brain regions, artifactual signals will tend to cancel one another out in the algorithmic equation.

We also hypothesize that, paradoxically, a possible mechanism for benefit of HIRREM could be the engagement between HIRREM and what is generally considered background noise or randomness in the EEG. The core technical aim of HIRREM is to resonate with dynamically changing dominant frequencies in the spectral EEG. Variations of amplitudes in these frequencies are typically characterized in stochastic terms. That is, *the energies of interest to HIRREM are in the category of apparently random fluctuations in the EEG, or noise*.

Variations in system noise levels can change the probability that a weak periodic signal will cross a threshold for sensory processing. The presence of an optimal noise level in a system can improve detection of a weak periodic signal, by boosting the signal sufficiently to cross the output threshold. For example, small increments in the luminance of a square presented to the right eye are better detected when there are tiny random fluctuations in the luminance of a square presented to the left eye than when the square presented to the left eye has constant luminance (Kitajo et al. 2003). This phenomenon has been referred to as stochastic resonance or stochastic facilitation, and it has been demonstrated for visual, auditory, and tactile sensory modalities (McDonnell and Ward 2011).

An implication of stochastic facilitation is that the system noise level may be a critical parameter for neural information processing (McIntosh et al. 2010; McDonnell and Ward 2011). If noise levels systematically change through HIRREM, it could be hypothesized that HIRREM impacts endogenous noise levels and thereby impacts overall efficiency of information processing.

### Possible contribution of placebo effects or other nonspecific factors

Delivery of HIRREM entails up to 10 or more visits (90 min each) with HIRREM technologists, instruction to relax while listening to musical tones, and being recumbent in a comfortable chair situated in a quiet environment. This combination of social interaction and relaxation induction might be predicted to produce improvements in self-reported well-being irrespective of the specific pitch or timing of musical tones produced through the HIRREM software algorithms. To establish definitively that clinical improvements associated with HIRREM are attributable to the specificity of software algorithms and not placebo effects or other nonspecific factors, placebo-controlled trials are indicated.

**Figure 6:**
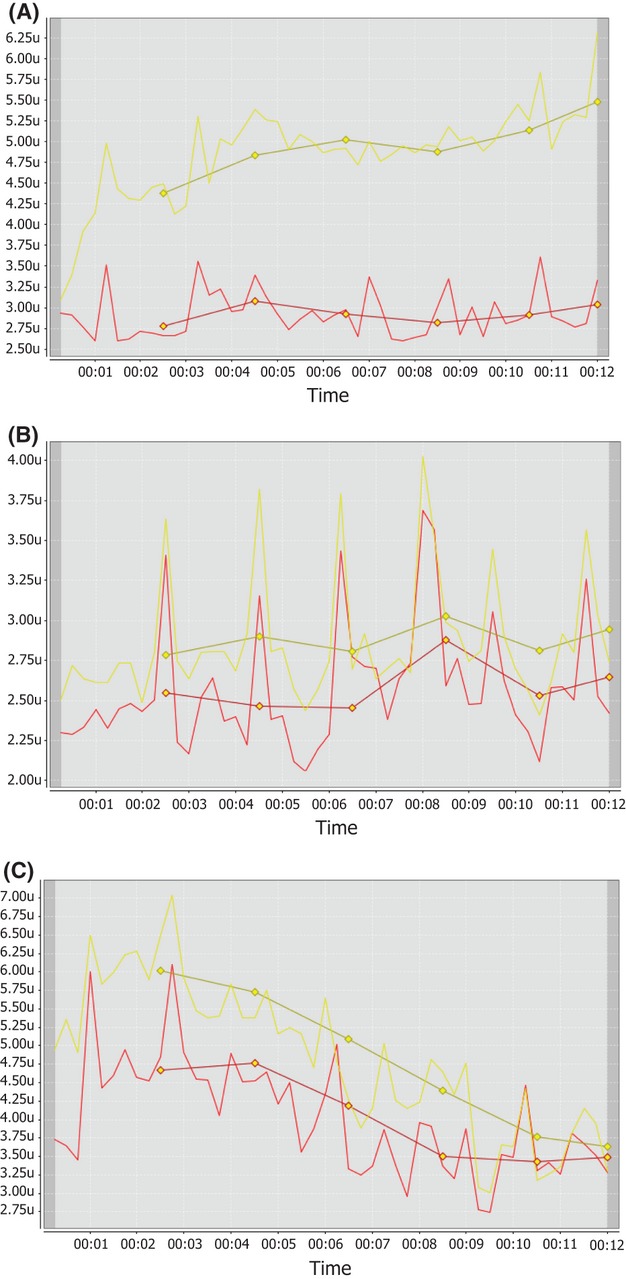
High-frequency (23–36 Hz) amplitudes (microvolts) in bilateral temporal lobes (T3 yellow, T4 red), for a 37-year-old man with insomnia, obtained from continuous EEG recordings (eyes closed) while listening to 12 min of white noise (A), random musical tones (B), and musical tones generated from HIRREM software algorithms (C).

As a preliminary illustration of the contrast between nonspecific relaxation induction and HIRREM, Figure 6 shows high-frequency (23–36 Hz) amplitudes in bilateral temporal lobes during exposure to three different types of sounds for a 37-year-old man with insomnia (Insomnia Severity Index Score 18, indicating moderate clinical insomnia) who presented to a community-based setting for HIRREM provision. Prior to beginning the standard HIRREM assessment and proceeding with the HIRREM intervention, the subject agreed to listen to three consecutive sets (12 min each) of “relaxing sounds“ while undergoing continuous EEG recording (using HIRREM technology as described in High-resolution spectral analysis of electroencephalic data and dynamic, iterative engagement of dominant frequencies). The first two sound sets were commercially available sound generators for white noise (http://www.simplynoise.com) and random musical tones (Winchime 3.0; http://www.sagebrush.com). The third sound set was a HIRREM protocol for the temporal lobes. In the interval before the second and third sound sets, the subject rested (1 min) and participated in a digit-recall task (1 min). Figures 6A and B demonstrate a consistent left hemispheric dominance while the subject listened to white noise and random musical tones, and no change in the amplitudes over the course of the sound sets. Figure 6C demonstrates gradual quieting, with progressive reduction of amplitudes beginning at minute 1 and continuing through minute 9, as well as disappearance of the asymmetry midway through minute 9, while listening to the HIRREM tones.

## Conclusion

Disturbances of neural oscillation have been reported with a variety of disease states, and there is a need for expansion of the repertoire of interventions which can positively impact oscillatory dynamics. The model of allostasis implies that brain functioning has consequences not only for neural systems but also for peripheral physiology, and thus further highlights the imperative for optimization of brain functional set points. Use of HIRREM, a noninvasive technology that creates sequences of resonance between neural oscillatory frequencies and musical tones, was associated with reduction of temporal lobe high-frequency asymmetry and fewer insomnia symptoms among individuals in a controlled clinical pilot trial. Studies are currently ongoing to further investigate potential applications of HIRREM and elucidate biophysical mechanisms of action.

## Supporting Information

**Table S1.** List of sequential tones for a sample HIRREM exercise.

**Audio File.** Sample of HIRREM musical tones (1 min).
