# Supplementary material for: HIRREM™: a noninvasive, allostatic methodology for relaxation and auto-calibration of neural oscillations
Source: Brain Behav. 2013 Jan 14;3(2):193–205. doi: 10.1002/brb3.116 (PMC3607159; doi:10.1002/brb3.116)
Supplement: Supplementary file 1 [file brb30003-0193-SD1.pdf]

Note List for Sample HIRREM Exercise

Note	Time
E4	10:55:12,767
E4	10:55:13,787
E5	10:55:14,730
E3	10:55:15,646
E4	10:55:16,471
C5	10:55:16,954
E5	10:55:17,431
A4	10:55:17,913
E4	10:55:18,557
G4	10:55:19,186
E4	10:55:19,965
A4	10:55:21,585
E4	10:55:22,079
E3	10:55:23,026
G2	10:55:24,164
G2	10:55:25,141
G2	10:55:25,891
C3	10:55:26,372
G2	10:55:28,560
E4	10:55:29,341
G4	10:55:30,782
G4	10:55:31,261
G2	10:55:32,300
E2	10:55:32,776
A2	10:55:33,256
E4	10:55:33,734
A2	10:55:34,548
E3	10:55:35,191
A2	10:55:36,331
A2	10:55:36,856
E2	10:55:37,620
E2	10:55:38,730
C4	10:55:39,347
E3	10:55:40,456
E3	10:55:41,671
C3	10:55:42,301
E3	10:55:42,961
G2	10:55:43,756
E3	10:55:44,356
E3	10:55:45,094
G2	10:55:45,991
E4	10:55:46,501
G4	10:55:46,981

Notes

***Note* Column :**
The number after the note letter represents the 'octave' of that particular note. For example, C4 is 'Middle C' on the piano keyboard. All notes for this exercise are being generated from a Pentatonic Major Scale in the Key of C. There are no notes that are 'sharp' or 'flat'.

***Time* Column :**
The number for the time represents the 'actual time' that the note was played. For example, the first note was played at 10:55:12,767 - which represents that particular note being played at 10:55 am & 12.767 seconds.

Additional Note :
In the accompanying audio and video demo files, the first note does not play until approximately 3 seconds from the beginning and the last note starts playing at approximately 3 seconds before the end.

A4	10:55:48,106
A3	10:55:49,034
E2	10:55:50,700
E4	10:55:51,345
E4	10:55:51,825
E3	10:55:52,935
E3	10:55:54,675
A3	10:55:55,740
E3	10:55:56,610
E3	10:55:57,090
A3	10:55:57,900
C3	10:55:58,995
A2	10:55:59,580
E3	10:56:00,914
A2	10:56:01,604
G4	10:56:02,205
E5	10:56:02,850
E3	10:56:03,330
E2	10:56:04,035
A2	10:56:04,875
E2	10:56:05,580
C3	10:56:06,059
E3	10:56:06,538
E2	10:56:07,369
E3	10:56:07,949
A2	10:56:08,623
